# Organoids as a Model for Precision Medicine in Malignant Pleural Mesothelioma: Where Are We Today?

**DOI:** 10.3390/cancers14153758

**Published:** 2022-08-02

**Authors:** Yanyun Gao, Marianna Kruithof-de Julio, Ren-Wang Peng, Patrick Dorn

**Affiliations:** 1 Department of General Thoracic Surgery, Inselspital, Bern University Hospital, University of Bern, 3008 Bern, Switzerland; yanyun.gao@dbmr.unibe.ch; 2 Department of BioMedical Research (DBMR), Oncology-Thoracic Malignancies (OTM), University of Bern, 3008 Bern, Switzerland; 3 Urology Research Laboratory, Department for BioMedical Research (DBMR), University of Bern, 3008 Bern, Switzerland; marianna.kruithofdejulio@dbmr.unibe.ch; 4 Department for BioMedical Research (DBMR), Translation Organoid Research, University of Bern, 3008 Bern, Switzerland; 5 Department of Urology, Inselspital, Bern University Hospital, 3008 Bern, Switzerland

**Keywords:** mesothelioma, organoids, tumor model, drug screens, precision medicine

## Abstract

**Simple Summary:**

Malignant pleural mesothelioma (MPM) is an extremely lethal cancer, notoriously known for its limited treatment options, lack of targeted therapies, and catastrophic survival rates. MPM tumors are highly heterogeneous and exhibit substantial variance in the genome landscape among individual patients, characterized by widespread loss-of-function mutations of tumor suppressor genes (TSGs) that are difficult to target. Therefore, there is an urgent and unmet need for novel therapeutic targets and strategies for personalized treatment. Patient-derived organoids (PDOs), the next generation tumor models that have significantly influenced the discovery of anticancer drugs and biomarkers of response to therapies in many other cancers, are emerging and promise to play a critical role in understanding the biology of MPM and, importantly, in identifying and developing precision oncology approaches tailored to specific subsets of MPM patients.

**Abstract:**

MPM is an aggressive tumor originating from pleural mesothelial cells. A characteristic feature of the disease is the dominant prevalence of therapeutically intractable inactivating alterations in TSGs, making MPM one of the most difficult cancers to treat and the epitome of a cancer characterized by a significant lack of therapy options and an extremely poor prognosis (5-year survival rate of only 5% to 10%). Extensive interpatient heterogeneity poses another major challenge for targeted therapy of MPM, warranting stratified therapy for specific subgroups of MPM patients. Accurate preclinical models are critical for the discovery of new therapies and the development of personalized medicine. Organoids, an in vitro ‘organ-like’ 3D structure derived from patient tumor tissue that faithfully mimics the biology and complex architecture of cancer and largely overcomes the limitations of other existing models, are the next-generation tumor model. Although organoids have been successfully produced and used in many cancers, the development of MPM organoids is still in its infancy. Here, we provide an overview of recent advances in cancer organoids, focusing on the progress and challenges in MPM organoid development. We also elaborate the potential of MPM organoids for understanding MPM pathobiology, discovering new therapeutic targets, and developing personalized treatments for MPM patients.

## 1. Introduction

Malignant mesothelioma is a rare but aggressive cancer type, arising from the mesothelium (the serosal outer linings) of the pleura, pericardium, peritoneum, and tunica vaginalis that cover the lung, heart, abdomen, and testes, respectively. Malignant pleural mesothelioma (MPM) accounts for 90% of all mesotheliomas, with the 5-year survival rate remaining 5% to 10% [1]. Exposure to asbestos is the most common cause of MPM with a latency period of 20 to 50 years [2]. Asbestiform fibers (erionite, winchite, magnesio-riebeckite, richterite, Libby asbestos, antigorite, and fluoro-edenite) causally relates to MPM [3]. Histologically, MPM is divided into four subtypes: epithelioid (50–60%), sarcomatoid (10%), biphasic (30–40%), and desmoplastic (<2%) [4], with epithelioid subtype associated with better survival compared with the other subtypes [1,5]. Molecularly, MPMs feature widespread mutations in TSGs, including BAP1, CDKN2A, and NF2, while driver mutations in oncogenes are rare, which poses a significant challenge for the development of targeted therapies against MPMs [6,7,8]. Platinum-based doublet chemotherapy is the standard first-line treatment for advanced MPM since 2003 [9], with effective second-line treatment that overcomes inevitable drug resistance still elusive [10]. Immunotherapy (e.g., immune checkpoint inhibitors, ICIs) has been recently approved as a new first-line treatment for unresectable MPM [11] because of the favorable benefit for patients compared with chemotherapy in clinical trials [11,12]. Consequently, novel therapeutic targets and strategies are urgently needed to effectively treat MPM [13,14,15,16]. Recent evidence reveals that MPM tumors are highly heterogeneous, which challenges one-size-fits-all strategies [17,18,19,20] and instead underscores the need for precision oncology-based personalized care of MPM patients.

Accurate preclinical models that faithfully recapitulate the genomic and histopathological features of MPMs are critical for identification and development of precision medicine [21]. Two-dimensional (2D) culture of MPM cell lines, established from primary tumors or pleural fluid [22], are the most-used models but have significant limitations, such as the lack of tissue architecture and complexity of in vivo biological processes [23]. Animal models of MPM have also been established, including asbestos-induced murine tumors, MPM-prone genetically modified mice, and patient-derived xenograft (PDX) models [24,25,26]. While 2D culture and the mouse models are useful, patient-derived organoids (PDOs), an in vitro culture of ‘organ-like’ three-dimensional (3D) structure that faithfully mimics the biology and complex architecture of the primary cancer, represent the next-generation tumor model with obvious advantages over other existing models [27]. Organoids have been successfully established in colorectal, gastrointestinal, pancreatic, prostate, liver, and brain cancers, but efforts to develop MPM organoids are still a largely unfulfilled endeavor [28,29,30]. In this review, we present recent advances in PDOs, with a focus on the progress and challenges in developing MPM organoids. We also discuss how MPM organoids will revolutionize our understanding of MPM pathobiology at the molecular level, facilitate the discovery of new therapeutic targets and strategies, and accelerate the development of personalized precision medicine for MPM patients.

## 2. Brief History and Current Status of Organoids

The term ‘organoid’ refers to mini-clusters of cells that self-organize and differentiate into functional cell types in vitro and recapitulate the structure and function of an organ in vivo (therefore, also called “mini-organs”) [31]. The organoid culture dates back to 1907, when H. V. Wilson first reported that sponge cells could self-organize to regenerate an entire organism in vitro [31,32]. A few decades later, researchers performed dissociation and re-aggregation experiments to generate different organs from stem cells of embryos in dishes [31,33]. With the development of stem cell research, such as the isolation of pluripotent stem cells (PSCs) and the generation of induced PSCs (iPSCs), organoid research progressed strikingly in the late 20th and early 21st centuries, as organoids can be generated from PSCs (embryonic and adult stem cells) and iPSCs [34,35,36]. In 2009, single leucine-rich repeat-containing G-protein-coupled receptor 5 (Lgr5)-expressing adult intestinal stem cells formed 3D intestinal organoids in Matrigel that self-organized and differentiated into crypt-villus structures without a mesenchymal niche; this was the first report of a 3D organoid culture derived from a single adult stem cell [37]. Since then, 3D organoid systems have attracted much attention and shown tremendous potential for modelling human cancers [38,39,40,41]. To date, organoids have been developed for many cancers, including colon cancer [42], gastrointestinal cancer [29], pancreatic cancer [43], prostate cancer [30,44], bladder cancer [45,46], liver cancer [47], breast cancer [48], and brain cancer [49].

## 3. PDOs in Cancer Research

There has been increasing interest in the development and utilization of patient-derived organoids (PDOs) for cancer research. Paralleled with this development, various PDO-derivation methods and protocols have been developed for different cancer types.

### 3.1. Methods for Establishing PDOs

There are now several methods for generating PDOs, including Matrigel-based culture, suspension culture, and culture on chips. Most human cancer organoids could be produced using Matrigel, which is a hydrogel at 24–37 °C and a liquid at 0–4 °C. Specifically, single cells taken from human tumor tissue are resuspended in Matrigel or Matrigel-containing organoid media [50]. The cultivation of organoids from different cancers differs in terms of the method of tissue digestion, density of seeded cells, Matrigel concentration, type of culture plate, and culture medium. Recently, various biomaterials have been developed as substitutes for Matrigel, as Matrigel cannot be readily tailored to create specific niches for organoids that are reminiscent of a particular organ [51]. Organoids can also be cultured as a suspension in a medium without Matrigel [52]. The key to suspension culture is the use of an ultra-low attachment plate whose surface is coated with a special hydrogel that prevents adsorption of extracellular proteins to the plate surface and minimizes adhesion of monolayer cells to the culture vessel. The formulation of the culture medium is also crucial for the successful culture of organoids in suspension. To mimic tissue–tissue interfaces, organ-level structures, fluid flow, and the mechanical effects to which cells in living organs are exposed, organoids on chips have also been developed to meet these requirements [53,54]. The ingredients of the medium are crucial for the generation of PDOs. Depending on the tissue type, different growth factors are required to stimulate organoid growth [38]. A ROCK (Rho-associated protein kinase) inhibitor was used at the beginning of culture to prevent anoikis [55]. With the development of new technologies, organoids of colorectal cancer could be generated from a single cell, allowing better definition of heterogeneity within the tumor [56,57]. A microdissection protocol to generate uniform, sub-millimeter glioma PDX tumor cubes has standardized the tissue mincing techniques for organoid cultures [58].

### 3.2. Applications of PDOs in Cancer Research

PDOs are invaluable tools for translational study as well as basic research [27,59], with drug screening and testing of personalized treatment among the best-studied cancers [30,46,60,61,62,63]. In addition, PDOs are increasingly being used to investigate the mechanisms of tumorigenesis [64,65,66,67,68], the tumor microenvironment (TME) [69], infection–cancer progression [69,70,71], cancer metastasis [71,72], and immunological cancer research [69,70,73,74].

## 4. Progress and Challenges in the Development of MPM Organoids

Despite the success of PDOs in many other cancers, the development of MPM organoids is still in its infancy. In this section, we describe recent progress and unresolved challenges in initiating MPM organoids.

### 4.1. Advances in MPM Organoid Development

The most commonly used MPM model is 2D culture of cell lines [75] established from primary tumors or pleural fluid, which can be easily cultured and manipulated but with significant limitations [21]. Recent attempts to overcome the limitations of 2D culture has led to the development of several 3D models of MPM (Table 1). 

In 2005, Kim et al. reported (Table 1) the culture of tumor spheroids from mesothelioma tissues that contained viable mesothelioma cells, macrophages, and a collagen-rich stroma at 37 °C in 5% CO_2_ with 100% relative humidity [76]. Three-dimensional spheroid cultures from MPM cell lines and ex vivo tumor fragments have also been reported [77,78,79,81,83,84]. In 2013, the Guenat group successfully grew multicellular spheroids from very low numbers of MPM cells in ultra-low attachment plates and loaded the spheroids into a microfluid platform to test sensitivity to chemotherapy [80]. However, these models do not significantly overcome the shortcomings of 2D culture because of the lack of tumor heterogeneity and TME, and the technical difficulty for long-term expansion or maintenance.

A breakthrough was achieved in 2018, when a study first reported that personalized organoids from two patients with epithelioid peritoneal mesothelioma were successfully generated using tumor-on-a-chip microfluidics and were suitable for drug screening [82]. In detail, fresh mesothelioma tissue samples (within one hour after surgery) were washed in phosphate buffered saline (PBS) with 2% penicillin-streptomycin for three 5 min cycles and then washed in Dulbecco’s Modified Eagle’s Medium (DMEM) with 2% penicillin-streptomycin for two 5 min cycles before dissociation with 10% collagenase/hyaluronidase for 18 h at 37 °C on a shaker. The digested tumor was filtered through a 100 µm cell filter and centrifuged to get a cell pellet, followed by removal of red blood cells. The isolated tumor cells were mixed with a photopolymerizable hyaluronic acid (HA) and gelatin hydrogel precursor at a density of 20 million cells/mL and placed in an adhesive film-based microfluidic device with multiple independent sets of channels. A tumor construct was biofabricated in each circular chamber of the device by ultraviolet light exposure (365 nm, 18 W cm^−2^) through an integrated photomask. Finally, the unexposed precursor/cell mixture was flushed from the device with PBS, and discrete 3D patient-derived mesothelioma constructs remained in each channel and were continuously supplied with DMEM media from independent reservoirs by tubing connected to a micro-peristaltic pump. The tumor organoids were confirmed to retain the mesothelioma phenotype and to be suitable for in vitro drug screening [82]. This was the first implementation of mesothelioma PDO culture, and the drawback is that special equipment is required. Although much remains to be explored to determine whether the protocol should be tailored to the needs of different histologic and molecular subtypes of MPM, this study signals the end of the darkness and possibly the horizon of the long-awaited in vitro 3D model of MPM of unprecedented clinical relevance.

### 4.2. Challenges in the Development of MPM Organoids

Despite the superiority of MPM PDOs over other 3D models, including ex vivo organotypic culture of tumor tissue MPM [76,77,78,79], there are challenges that have hindered the broad application of the model in translational research. First, it is still unclear how to optimize culture conditions (e.g., media, growth factors) to enable the culture and expansion of MPM organoids from primary tumors with different histological and genetic profiles [85]. Patient-derived pleural fluid can be used to generate MPM organoids, as pleural fluid management is a common clinical problem in MPM patients. Pleural fluid has been reported to promote the proliferation of cancer cells with pro-growth biological properties [86]. Second, the high secretion of MPM cells may destroy the solidified Matrigel before organoids mature. Third, the integrity of TME in organoids remains a problem in almost all types of tumor organoid models, including MPM [87].

## 5. PDOs and Precision Medicine for MPM

**Figure 1:**
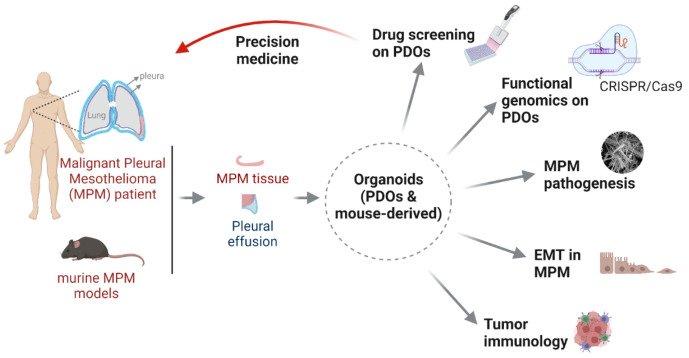
Patient-derived organoids as model of precision medicine for MPM.

The heterogeneity of MPM tumor subpopulations [88,89] has led to the consensus call for the application of precision oncology to MPM, and PDOs provide an unprecedented platform for identifying and developing precision medicine strategies for this daunting disease (Figure 1).

Treatment options for MPM are extremely limited, and patients do not have access to target therapies. Therefore, platinum-based chemotherapy, approved by the FDA in 2004, remains the standard of care. Recently, immunotherapy has also been approved, but only a fraction of MPM patients respond to treatment [86]. Identification of the molecular mechanisms underlying MPM pathogenesis and response to existing therapies promises to guide future development of precision medicine for MPM.

### 5.1. Personalized PDOs for Modelling MPM Heterogeneity

Molecular gradients, a measure of intra-tumor heterogeneity and of high prognostic value for patients, have recently been shown to improve MPM classification treatment [90]. Importantly, genetic alterations in TSGs stratify MPM patients into distinct groups that not only differ in molecular pathogenesis but also in therapy responses [67]. To precisely represent MPM heterogeneity, personalized PDOs are needed to model the disease for understanding the biology of the tumor and identifying precision oncology approaches [67]. Given the high fidelity of PDOs that recapitulate tumor heterogeneity cancers [28,49,91], a personalized PDO biobank of MPM that is amenable to translational and basic studies will provide unprecedented insights into the biology and therapeutic vulnerabilities of MPM (Figure 1).

### 5.2. MPM PDOs for Drug Screening

PDOs of many other cancers rates [92] have proven useful for drug screening and testing. Organoids of liver cancer are able to predict drug sensitivity or resistance in a patient-specific manner [47], as are lung cancer PDOs, which allow profiling of cancer patients’ response to drugs within a week [93].

A high-throughput screen of 2427 drugs using tissue-originated spheroid (CTOS), an ex vivo model from PDX tumors, was performed in colorectal cancer [62,94]. The automated devices—an organoid handler and a reagent dispenser—were used for this high-throughput screening. In order to obtain more tumor material for organoid culture, PDX tumors were used to generate organoids for drug screening in various cancers [91,95]. In ovarian cancer, it has been reported that patient-specific genomic alteration correlates with drug effects in organoids but not in 2D cell monolayers, suggesting that 3D organoids are a better model than 2D primary cells [96]. It may be necessary to add a 2D model, if possible, when drug testing is performed with organoids to show superiority in actual situations.

Although drug screening with MPM PDOs has not yet been reported, personalized PDOs will be of particular importance for unbiased genetic and pharmacological studies to discover novel anti-MPM therapies in a manner tailored to individual patients (Figure 1). The fact that PDOs allow high-throughput drug screening will facilitate the subsequent selection of the most efficacious drug to treat MPM. An important consideration for such screening is to enable high-throughput drug testing, which requires multiple passages and the long-term culture of organoids, as has been described in lung and other cancers [97]. In a recent study, culture conditions for PDOs suitable for large-scale drug screening were systematically investigated [61].

The concept of drug repurposing has attracted considerable attention [62,98]. Under this framework, FDA-approved drugs are evaluated for their efficacy against cancer. The same concept can be applied to the identification of drug combinations that have been shown to be an effective strategy to overcome treatment resistance, as we have recently demonstrated [10]. Advances in high-throughput screening systems also enable rapid analysis of large numbers of drug compounds using automated machines to dispense cells and drugs, and to perform endpoint measurements [99].

### 5.3. MPM PDOs for Functional Genomics

The CRISPR/Cas9 gene editing system is a powerful platform for functional genomics, which has been successfully used for genome editing of colorectal cancer organoids [100] and other organoid models [66,101,102]. In particular, gene knockout in tumor organoids using CRISPR/Cas9 provides functional evidence for the main drivers of oncogenes in colorectal cancer and can be used to validate various therapeutic approaches [64]. The suitability of PDOs for functional genomics suggests that they can serve as clinically relevant models for MPM and enable unprecedented investigations to discover novel therapeutic targets and vulnerabilities, as well as strategies for developing precision medicine to treat MPM (Figure 1).

### 5.4. MPM PDOs for Other Applications

MPM PDOs are also useful for studying fundamental mechanisms of tumor development, progression, resistance to cancer therapies, and TME (Figure 1).

Asbestos exposure is the major risk factor for MPM, but the mechanism underlying asbestos oncogenesis has not been fully understood [103]. Mouse models for asbestos-induced MPM have been developed, but the genetic profile is different from that of MPM patients because BAP1, NF2, or LATS2, which are frequently mutated in MPM patients, are not present in these mouse models [104]. Therefore, new models are needed to better understand the development and progression of MPM, and organoids derived from normal pleura may be a good option to study the pathogenic role of asbestos and to model the pathophysiology of MPM (Figure 1). Such organoids can be obtained from normal pleura or other autologous sources, such as iPSCs, as considerable progress has been made in the preparation of organoids from normal tissue [105,106]. Moreover, PDOs can be subjected to CRISPR/Cas9-mediated genomic editing to explore the molecular mechanisms underlying MPM out-growth, clone evolution, and drug resistance, as demonstrated in other cancers [65,101,102,107].

Epithelial-to-mesenchymal transition (EMT) plays a crucial role in MPM development, progression, and resistance to therapy [108,109], with the underlying mechanisms and key regulators largely unknown. As organoids are accessible to pharmacological and genetic perturbations, PDOs are a promising model to study the roles of EMT in MPM (Figure 1).

Cancer cells actively and dynamically interact with the TME and this reciprocal interaction significantly influences tumor progression and drug response. MPM is known to have a tumor-promoting TME due to chronic inflammation. Immunotherapy has recently been approved by the FDA for advanced MPM, whereas unselected patients respond very differently to this therapy. Therefore, it is critical to understand the underlying mechanism of response or resistance to therapy to prospectively stratify subgroups of patients who will benefit from immunotherapy. With advances in organoid culture technology, the incorporation of immune components has been increasingly recognized and realized [72]. The TME of the original tumors can be modeled using air–liquid interface PDOs or microfluidic devices [53]. Alternatively, the TME can be reconstituted by adding purified immune populations from original tumors or peripheral blood into submerged tumor organoids [69]. Consequently, PDOs can be exploited to study not only cancer-cell-intrinsic mechanisms but also the dynamic interplay between cancer cells and the TME (Figure 1).

## 6. Concluding Remarks and Prospective Directions

Despite the consensus call for the application of precision oncology in MPM, this disease continues to be approached both clinically and preclinically with one-size-fits-all strategies that fail to leverage the marked heterogeneity between patients. This is due, in part, to the lack of clinically relevant tumor models amenable to precision oncology approaches. PDO has emerged as an important platform to address clinically relevant questions in precision oncology of cancer, and ongoing efforts to model MPM with PDO are active, which holds the promise to accelerate the discovery of new, personalized treatments for the disease. An important prerequisite for accelerating precision medicine with MPM PDOs is standardization of PDO culture and drug screening. The timing of the procedure will also be critical to obtain information on drug sensitivity of PDOs from drug screening. However, as with any tumor model, there are limitations with organoids. First, culturing an organoid is both time- and resource-consuming compared to other cancer models [27]. Another dramatic limitation of organoid models is the lack of a vascularization system, despite recent attempts to overcome this drawback by using microfluidics or co-culturing with endothelial cells [110,111]. Finally, it is still challenging to include all cellular components of the TME in PDOs, highlighting the need for further improvements. Nevertheless, the continued scientific effort to integrate tumors and their inherent TME into PDO models will revolutionize precision medicine and raise unimagined hopes for cancer patients.

## Figures and Tables

**Table 1:** Three-dimensional culture of mesothelioma.

Culture Type	Culture Device	Medium	Starting Material	Ref.
Spheroids	10-cm plate coated with 0.8% agar	DMEM, and LHC-MM	MPM patient tissues	[76,77,78,79]
Spheroids on a microfluid platform	Ultra-low attachment flat-bottom 24-well plate	RPMI-1640 media based	MPM cell line H2052	[80]
3D tumor spheres	Ultra-low attachment plate	MammoCult™ Human Medium	MPM cell lines	[81]
Ex vivo organotypic culture	Ultra-low attachment plate	DMEM with 20% FBS	MPM PDX tumor slices	[81]
PDOs	Microfluidic device	DMEM	Tumors from patients with peritoneal mesothelioma	[82]

Three-dimensional cultures of mesotheliomas. DMEM, Dulbecco’s Modified Eagle Medium; LHC-MM, Laboratory of Human Carcinogenesis- Minimal Medium; RPMI-1640, Roswell Park Memorial Institute 1640 Medium; MPM, Malignant Pleural Mesothelioma; 3D, Three-Dimensional; FBS, Fetal Bovine Serum; PDX, Patient-Derived Xenograft; PDOs, Patient-Derived Organoids.

## Data Availability

This study did not report any data.
